# Effect of stimulus width on simultaneous contrast

**DOI:** 10.7717/peerj.146

**Published:** 2013-09-05

**Authors:** Veronica Shi, Jie Cui, Xoana G. Troncoso, Stephen L. Macknik, Susana Martinez-Conde

**Affiliations:** 1 Department of Neurobiology, Barrow Neurological Institute, Phoenix, AZ, USA; 2 Unité de Neuroscience, Information et Complexité (CNRS-UNIC), Gif-sur-Yvette, France; 3 Department of Neurosurgery, Barrow Neurological Institute, Phoenix, AZ, USA

**Keywords:** Simultaneous contrast, Brightness, Size, Width, Illusion, Edges

## Abstract

Perceived brightness of a stimulus depends on the background against which the stimulus is set, a phenomenon known as simultaneous contrast. For instance, the same gray stimulus can look light against a black background or dark against a white background. Here we quantified the perceptual strength of simultaneous contrast as a function of stimulus width. Previous studies have reported that wider stimuli result in weaker simultaneous contrast, whereas narrower stimuli result in stronger simultaneous contrast. However, no previous research has quantified this relationship. Our results show a logarithmic relationship between stimulus width and perceived brightness. This relationship is well matched by the normalized output of a Difference-of-Gaussians (DOG) filter applied to stimuli of varied widths.

## Introduction

Perceived brightness of a stimulus depends on the background against which the stimulus is set, a phenomenon known as simultaneous contrast (Chevreul, 1839; Hess & Pretori, 1894; Heinemann, 1955; Hurvich & Jameson, 1966). The German physiologist Ewald Hering, considered the father of simultaneous contrast research, provided several–now classical–demonstrations of this effect (Hering, 1872; Hering, 1964; Hurvich & Jameson, 1966). Subsequent studies found simultaneous contrast to depend on the size of the test stimulus and the inducing field (Diamond, 1955; Stevens, 1967; Kitterle, 1972; Yund & Armington, 1975; Blakeslee & McCourt, 1997), with the strongest perceived effects occurring for small test stimuli (Marsden, 1969). No research has quantified the precise relationship between stimulus width and perceived brightness, however. Here we measured the relationship between simultaneous contrast and test stimulus width in a two-alternative forced-choice task. Quantified studies of simultaneous contrast provide a means to further our understanding of brightness perception and its underlying neural mechanisms.

### Present research in the context of previous studies of simultaneous contrast

**Figure 1:**
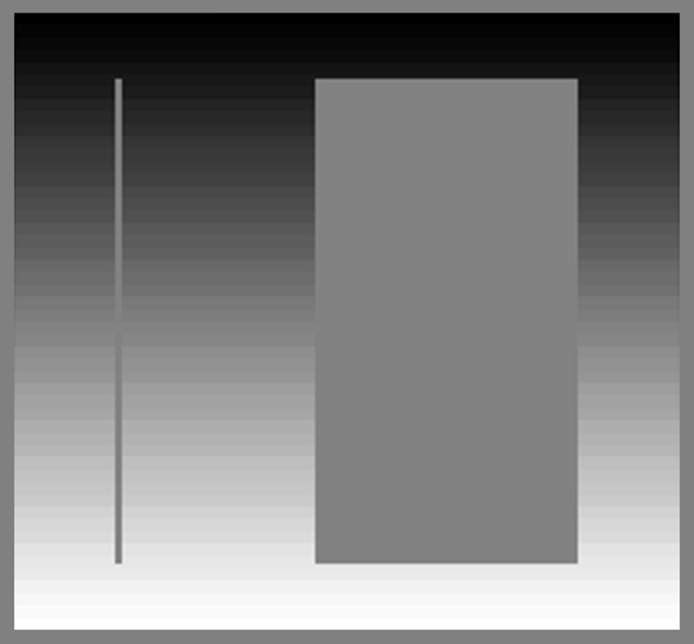
Effect of stimulus width on simultaneous contrast. The two bars are the same shade of gray throughout, but they appear lighter on the top (against a dark background) than on the bottom (against a light background). The narrow bar has a stronger effect on perception than the wide bar.

Michel Eugene Chevreul described simultaneous contrast in 1839 (Chevreul, 1839), and Hess and Pretori later tested it systematically (Hess & Pretori, 1894). Figure 1 illustrates simultaneous contrast with two test stimuli of different widths. Both test stimuli are the same shade of gray throughout, but they appear lighter on the top (against a dark background) than on the bottom (against a light background).

The strength of simultaneous contrast varies according to the characteristics of the test stimulus, such as its luminance (Diamond, 1953; Heinemann, 1955), spatial frequency content (Perna & Morrone, 2007; Shapiro & Knight, 2008), size (Diamond, 1953; Stevens, 1967), and proximity to the inducing field (Leibowitz, Mote & Thurlow, 1953). Previous studies found that brightness contrast effects increase with the size of the inducing field (Stevens, 1967; Yund & Armington, 1975). Diamond (1962) reported that test field area did not affect perceived brightness, but he used nearby, rather than surrounding, inducing fields (unlike in the present research). Studies using adjacent or nearby inducing fields or backgrounds that completely surrounded the test field found the greatest effects on perception for small test fields with large inducing surrounds (Yund & Armington, 1975; Blakeslee & McCourt, 1997); see also (Brenner & Cornelissen, 1991) for the effects of distances between boundaries on color vision. Here we presented test stimuli against a background comprising a luminance gradient (see also Shapiro & Knight, 2008; Geier & Hudák, 2011), so as to measure the inducing effects of both a dark and a light background with the same set of stimuli. The use of a common inducing gradient for brightness and darkness enhancement had the technical advantage of maintaining the overall luminance of the screen at 50% (see Methods for details), therefore controlling for the potential effects of luminance on pupillary responses (because the same number of photons entered the eye in all experimental conditions).

The current experiment set out to: (1) provide the first quantification of the relationship between stimulus width and simultaneous contrast by measuring the psychometric curves and then calculating the point of subjective equality (PSE) for each condition, and (2) compare the trends of perceptual responses to the trends from modeled responses from Difference-Of-Gaussians (DOG) filters. Whereas previous studies relied on a method of adjustment without any type of eye-movement monitoring, here we used a two-alternative forced choice discrimination task with strict monitoring of eye position.

Our results showed a logarithmic relationship between perceived brightness and test stimulus width, which was well matched by the normalized output of a Difference-of-Gaussians (DOG) filter applied to stimuli of varied widths, and a greater induction effect for brightness than for darkness perception.

## Methods

### Subjects

Seven adult subjects with normal or corrected-to-normal vision (6 females, 1 male; 4 naïve subjects, 3 authors) participated in these experiments. Each subject participated in 4 full experimental runs, each split into two 1-h sessions, and was paid $15 per session. Experiments were carried out under the guidelines of the Barrow Neurological Institute’s Institutional Review Board (protocol number 04BN039) and written consent was obtained from all subjects.

### Experimental design

Experimental details are similar to those in Troncoso, Macknik & Martinez-Conde (2005). Subjects viewed all stimuli binocularly, while resting their heads on a chinrest, 57 cm from a linearized video monitor (Barco Reference Calibrator V). We monitored their eye positions with a video-based eye tracker (Eyelink II; SR Research).

**Figure 2:**
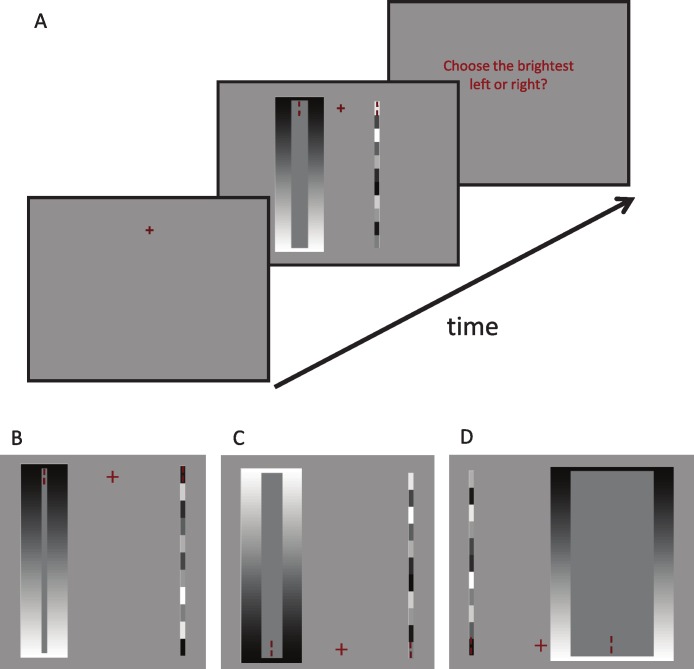
Psychophysical design. (A) Monitor display during the time course of a single trial. (B–D) Three different stimulus presentations of the brightness discrimination task (out of 1,584 possible combinations, see Methods section for details). The cross indicates the fixation point, and vertical red lines indicate the points to compare in the comparator and standard stimuli. Drawings not to scale.

To measure the magnitude of the perceptual effects, we conducted a two-alternative forced-choice brightness discrimination task between simultaneous contrast stimuli (i.e., comparator stimuli) and solid patches of a given shade of gray (i.e., standard stimuli), on a 50% gray background. At the beginning of each trial, subjects fixated a central red cross (1° within a 3.5° fixation window) and two sets of peripheral stimuli appeared simultaneously: the standard and the comparator (one centered at 7° to the left and one centered at 7° to the right of the fixation cross, see Fig. 2).

The comparator was a uniform gray bar against a 50-step luminance gradient that extended 2° on both sides of the bar. The bar had one of six possible widths (0.25°, 0.5°, 1°, 2°, 4°, and 8°) and one of three possible luminances (40%, 50%, or 60% gray). We used three different bar luminances so that subjects might not conclude that there was a single luminance in all conditions, and have this knowledge potentially influence their perceptual reports.

The standard strip was made of 11 luminance segments (with luminances of 5%, 14%, 23%, 32%, 41%, 50%, 59%, 68%, 77%, 86%, and 95%), pseudorandomly scrambled. Both the standard and the comparator stimuli subtended 18° vertically. Vertical red lines were displayed 0.55° from the top or bottom end of both standard and comparator to indicate the precise regions of the stimuli to be compared. The vertical lines could select any of the 11 possible luminance segments in the standard stripe with equal likelihood, and were always aligned with the center of one of the luminance segments. After two seconds, all stimuli disappeared.

The subjects’ task was to compare the brightness of the region positioned precisely in the center between the inner ends of the red lines on the standard stimulus, to the brightness of the same point on the comparator stimulus. Thus the region of interest in the comparator was compared against all possible luminances of the standard, for all widths tested.

The physical difference between comparator and standard was always a function of the luminance of the segment within the standard stimulus at the point of comparison. Therefore, if the comparator appeared brighter or darker than a standard segment of the same luminance, this was a perceptual effect, as there was no physical difference.

Half of the subjects (*n* = 4) indicated which stimulus appeared brighter at the discrimination point (the comparator or the standard) by pressing the left/right keys on the keyboard. To control for bias, the rest of the subjects (*n* = 3) indicated which stimulus appeared darker. The experiment design was controlled for other criterion effects as well, by giving subjects a bright-appearing comparator in one half of the trials, and a dark-appearing comparator in the other half of the trials. Also, the comparator was presented half of the time on the left, and half of the time on the right. The fixation cross was presented half the time on the top of the screen, and half the time on the bottom, with the bright half of the background on the upper half of the comparator half the time. Figure 2 shows several examples of comparators and standards.

Subjects did not need to wait until the stimuli turned off to indicate their decisions, but could answer as soon as they were ready, in which case the stimuli disappeared from the screen and the trial ended. If a subject broke fixation (as measured by Eyelink II), the trial was aborted, and replaced in the pseudorandom trial stream to be re-run later.

The study included 396 experimental conditions:

• 6 comparator widths: 0.25°, 0.5°, 1°, 2°, 4°, 8°
• 3 comparator luminances: 40%, 50%, 60%
• 2 inducing gradient luminances at the point of comparison: bar against the dark part of the gradient and bar against the bright part of the gradient.
• 11 standard luminances: 5%, 14%, 23%, 32%, 41%, 50%, 59%, 68%, 77%, 86%, 95%

We used 4 stimuli configurations to prevent potential bias:

• 2 screen positions: left and right
• 2 fixation cross locations: top and bottom

Each experimental session included 4 repetitions of each experimental condition (one for each stimulus configuration), amounting to a total of 1,584 combinations of experimental condition and stimulus configuration. Each subject participated in 4 full experimental runs (8 sessions); thus, each subject viewed each combination a total of 16 times.

**Figure 3:**
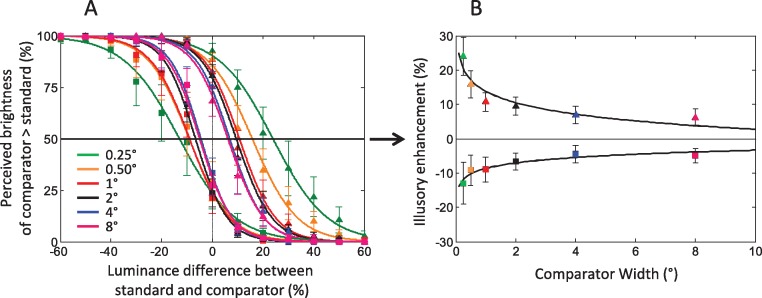
Psychophysical results. (A) Psychometric functions for the different stimulus widths are displayed in different colors. The conditions in which the comparator appears bright are indicated by triangles, and the conditions in which the comparator appears dark are indicated by squares. (B) Perceived enhancement of the PSEs for the different stimulus widths, with respect to the physical luminance of the comparator. The perceived enhancement of brightness (triangles) and darkness (squares) perception decreases as the stimulus width increases, approximately following a logarithmic function. Error bars in (A) and (B) represent the ± SEM for all subjects in each condition.

The independent variable was the luminance difference between standard and comparator, so we averaged together all combinations of comparator luminances and standard luminances that resulted in the same approximate difference in luminance (i.e., pair comparator 40%-standard 59% and pair comparator 60%-standard 77%, for a difference in luminance of 18% + /− 1%) (Fig. 3A).

### Center–surround simulations

To test the applicability of center–surround receptive fields and the lateral inhibition process as an explanation for the effect of stimulus width on the strength of simultaneous contrast, we modeled the process using Difference-Of-Gaussian (DOG) filters (Rodieck, 1965; Enroth-Cugell & Robson, 1966). The DOG filter was defined as:

\begin{eqnarray*} \displaystyle \text{Receptive-field}~(x,y)=\text{Center}~(x,y)-\text{Surround}~(x,y)={k}_{c}{e}^{\frac{-({x}^{2}+{y}^{2})}{2{r}_{c}^{2}}}-{k}_{s}{e}^{\frac{-({x}^{2}+{y}^{2})}{2{r}_{s}^{2}}},&&\displaystyle \end{eqnarray*}

where *r_c_* and *r_s_*, or the ‘radius’ of the center and surround respectively, represent the distance over which the sensitivities of center and surround fall to $1/\sqrt{e}$ of the peak value; *k_c_* and *k_s_* represent the peak sensitivities of center and surround (Troncoso, Macknik & Martinez-Conde, 2005). We defined the 2-D integrations of center (*x*, *y*) and surround (*x*, *y*), ${w}_{c}=2 \pi {r}_{c}^{2}{k}_{c}$ and ${w}_{s}=2 \pi {r}_{s}^{2}{k}_{s}$, as the weights (or volumes) of center and surround towards the filter output.

We generated gray bars of 50% luminance with different widths against a black-to-white gradient, and then convolved them with the DOG filter. The output of the convolution simulated the activity of an array of retinotopically-arranged center–surround neurons looking at the image. We compared this output to the empirical perceptual results.

### Data fitting

**Figure 4:**
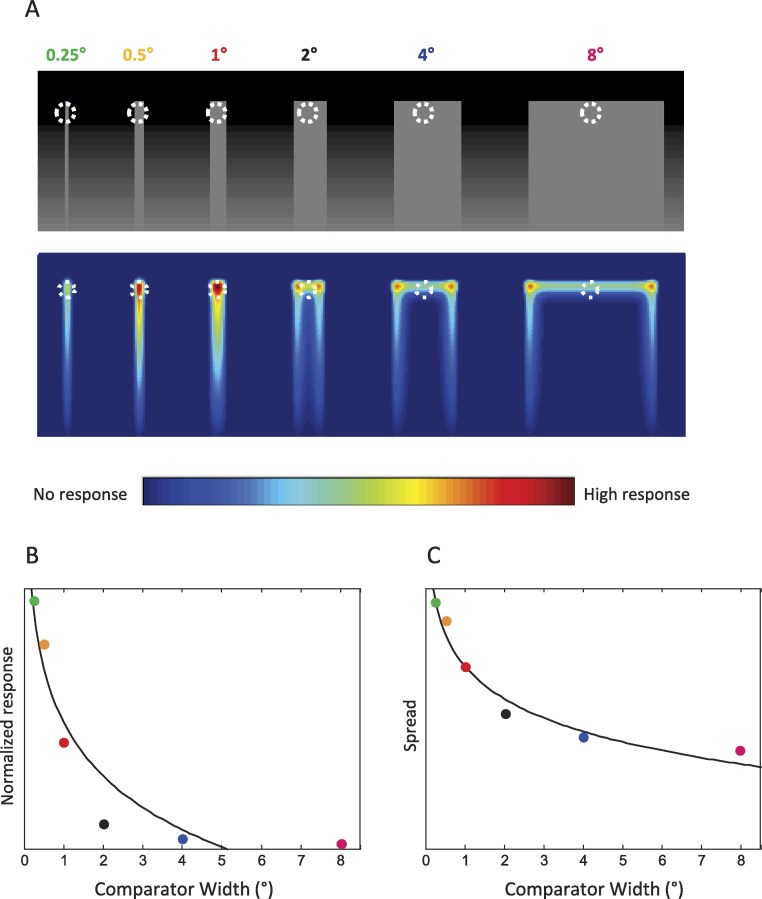
Computational simulations with a DOG filter. The filter parameters were chosen to match physiological center–surround receptive fields at the eccentricity used in the psychophysical experiments (7°). (A) Top: Examples of stimuli analyzed in the simulations (top half of dark-to-bright gradients). The six different comparator widths are illustrated. These stimuli were equivalent to the comparators presented in the psychophysical experiment. The white dashed circles denote the regions of comparison during the psychophysical experiments. Bottom: Predicted responses from a DOG filter. Convolving the DOG filter with the stimuli at the top simulates the output of an array of center–surround neurons. (B) Normalized responses, at the point of discrimination in the psychophysical experiment, for each width. For each data point, the response was divided by the bar width. (C) Spread of the response for each stimulus width. We added the responses along the width of each bar, at the height of discrimination, and divided the total by the bar width. Data points indicate the widths used in the psychophysical experiment. Black lines indicate the fits to the logarithmic function used to fit the data in Fig. 3B.

**Figure 5:**
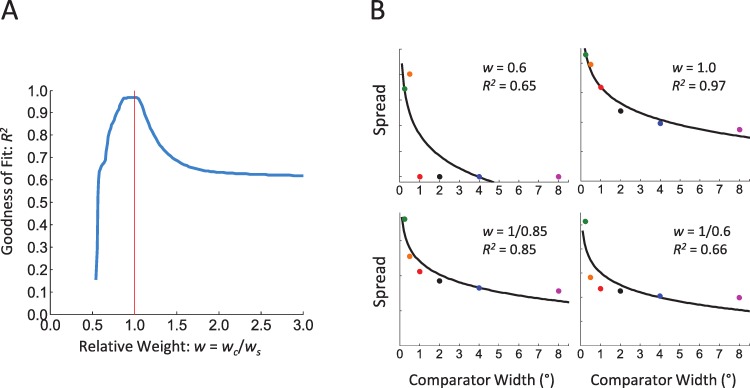
Influence of the relative weights of the DOG model’s center and surround on data fitting. (A) Goodness of fit (*R*^2^) as a function of relative weight (*w*). The red vertical line indicates equal weight of center and surround (*w* = 1). Note that only the results corresponding to *w* ≥ 1 are biologically relevant. We show the results for *w* < 1 for mathematical exploration. There was no correlation between spread and logarithmic function for *w* ≤ 0.5. (B) Examples of data fitting for different *w* values. Upper Left: spread and fitted curve when the weight of the center is 60% of that of the surround (*w* = 0.60). Upper Right: equal weights of center and surround (*w* = 1, i.e., Fig. 4C). Lower Left: the weight of the surround is 85% of that of the center. Lower Right: the weight of the surround is 60% of that of the center.

Psychometric curves (Fig. 3A) were obtained by fitting the data with logistic functions using a maximum likelihood procedure (Wichmann & Hill, 2001). We used Matlab’s nonlinear curve-fitting with the Levenberg–Marquardt algorithm (Matlab’s Curve Fitting Toolbox; MathWorks, Inc.) to fit the data in Figs. 3B, 4 and 5.

## Results

### Psychophysical test

We found that simultaneous contrast was enhanced for narrow stimulus widths and weakened for wide stimulus widths. To objectively quantify the strength of the effect, we calculated the point of subjective equality (PSE) for each comparator (i.e., its matching luminance in the standard) by determining the point on the psychometric curve (Fig. 3A) in which the comparator appeared more salient than the standard in 50% of the trials. We averaged the responses of all subjects, and collapsed across the following conditions: comparator luminance (40%, 50%, and 60% gray), and point of discrimination (top or bottom). We expressed the results as a function of the physical difference in luminance between standard and comparator, and calculated the perceived enhancement for each comparator width as the difference between the PSE for the width tested and its actual physical luminance (Fig. 3B).

Perceived brightness varied parametrically with stimulus’ width, with narrow bars generating stronger perceptual effects than wide bars. At the two extremes, the narrowest (0.25° width) and widest (8° width) comparators produced perceived enhancements of ∼30% and ∼ 8%. We found a greater effect for brightness than darkness induction, in agreement with Blakeslee & McCourt (1999). Further, there was a logarithmic relationship (fitting function: *f*(*x*) = *a* + *b*log(*x*)) between perceived brightness and stimulus width (Fig. 3B), which had not been reported previously.

### Center–surround simulations

Center–surround receptive fields and lateral inhibitory processes have been proposed as a potential explanation for various brightness illusions (Macknik, Martinez-Conde & Haglund, 2000; Troncoso, Macknik & Martinez-Conde, 2005; Troncoso et al., 2007; Troncoso, Macknik & Martinez-Conde, 2009). Here we modeled center–surround receptive fields as DOG filters (Rodieck, 1965; Enroth-Cugell & Robson, 1966), matching their size to the range of physiological center–surround receptive fields in the primate (Derrington & Lennie, 1984; Irvin, Casagrande & Norton, 1993; Tadmor & Tolhurst, 2000; Levitt et al., 2001) at the eccentricity used during the psychophysical experiments (7°), see Methods for details. Both center and surround had the same weight towards the DOG filter’s output, i.e., *w_c_* = *w_s_*; thus the filter’s output in response to uniform luminance was zero. Figure 4 shows the results of convolving a DOG filter (*r_c_*: 0.25°; *r_s_*: 0.50°) with the varying width comparators (top halves) presented during the psychophysical experiments.

The predicted strength of the effect varied in correlation to the comparator width, with narrow stimuli generally producing stronger outputs (Fig. 4A). There were some discrepancies between the filter’s output and the psychophysical results, however. Whereas the psychophysical results showed a parametrical relationship between increased brightness perception and decreasing stimulus widths (Fig. 3), the modeling results indicated a maximum response for a stimulus width of 1° (Fig. 4A). Further, the modeling results indicated little or no response at the point of discrimination in the wider bars. To account for these differences, we normalized the DOG filter responses in two alternative ways:

The first method consisted of a simple normalization (i.e., we divided the response at the point of discrimination, 0.55° from the top of each bar, by the bar width). The results, in Fig. 4B, showed a smoother relationship between stimulus width and predicted perceived strength, but did not capture the perceptual effects for the wider widths (4° and 8°), for which the model’s output was still minimal. Thus, we implemented a second method of normalization, to represent the “spread” of the perceptual response over the entire width of the stimulus. Thus, for each stimulus, we summed the model’s output along the stimulus’ width at the discrimination height and then divided it by width. Blakeslee & McCourt (1999) used a somewhat similar approach, by averaging the output of their DOG model across the width of their test patches to arrive at a single-valued prediction. Figure 4C shows the output of this spread function, which now resembles the psychophysical curve (Fig. 3B). This method of normalization produces substantial responses for the wide conditions, in agreement with the psychophysical data. Thus, a DOG model’s normalized output may adequately predict the relationship between stimulus width and perceived strength of simultaneous contrast.

### Influence of relative weights of center and surround

To determine the importance of the relative weights of our DOG model’s center and surround, $w=\frac{{w}_{c}}{{w}_{s}}$, to the fitting, we next varied *w* and measured the goodness of fit to the logarithmic function determined by the empirical data (Fig. 3).

The relative weights of center and surround were critical to the goodness of fit (Fig. 5). Equal weights (*w* = 1) resulted in the best fit. We also observed a suitable fit for *w* = 1/0.85 = 1.18 (i.e., with the weight of the surround being 85% of that of the center), a value that represents experimental findings for cat and monkey retinal ganglion cells and lateral geniculate nucleus neurons (Tadmor & Tolhurst, 2000). In this condition, the normalized output of the DOG filter still adequately predicted the relationship between stimulus width and brightness enhancement (*R*^2^ = 0.85), thus capturing the essential characteristics of the psychophysical response.

## Discussion

Our results are in general agreement with those of previous studies (Yund & Armington, 1975; Blakeslee & McCourt, 1997) in that a decrease in test stimulus size leads to an increase in simultaneous contrast. In addition to previous studies, we found a logarithmic relationship between test stimulus width and perceived brightness. This function gradually levels off, indicating that there may not be one distinct threshold or point after which simultaneous contrast ceases to exist. This is supported by the observation that brightness induction can occur in test fields as large as 10° (Yund & Armington, 1975). Because this width is much greater than the size of early visual receptive fields in monkeys (De Valois & Pease, 1971; Yund et al., 1977), filling-in processes may contribute to the brightness of the test field (Paradiso & Nakayama, 1991).

### Center–surround receptive fields and lateral inhibition

Early visual neurons respond more strongly to local contrast changes than to regions of uniform luminance (Marr & Hildreth, 1980; Troncoso, Macknik & Martinez-Conde, 2005; Troncoso et al., 2007; Troncoso, Macknik & Martinez-Conde, 2009). Thus the perception of simultaneous contrast may rely on the properties of early center–surround receptive fields and lateral inhibitory processes (Diamond, 1960; Thomas, 1970).

The DOG simulations’ normalized output showed a logarithmic relationship between predicted responses and stimulus’ width (Figs. 4B and 4C) in agreement with our psychophysical results (Fig. 3B). The non-normalized DOG responses (Fig. 4A) correlated with perception in general, though they showed substantial discrepancies with the psychophysical observations, suggesting that simple (i.e., non-normalized) DOG linear filters do not completely explain the perception of simultaneous contrast. Normalizing with the “spread” of the responses adequately captures the logarithmic relationship between stimulus width and perceived brightness that we found in the psychophysical data, suggesting an underlying filling-in mechanism that takes into account both the responses to the edges and to the inside region of the test stimulus.

The present observation of a stronger brightness than darkness enhancement effect is consistent with the results found by Blakeslee & McCourt (1999). Although Blakeslee and McCourt’s model did not capture the asymmetry, the authors proposed that the application of different gain parameters to the outputs of independent ON- and OFF-channels would be a logical first step towards accommodating the perceptual differences observed. Such modification of the model might be in line with the physiological effects of lateral activation and inhibition of ON-center neurons versus OFF-center neurons.

Our combined psychophysical and modeling results suggest that simultaneous contrast is not fully accounted for by linear center–surround receptive fields, but may also involve filling-in or higher-level cortical processes. Further, receptive field size is known to vary with retinal eccentricity (Hubel, 1995), and so future research should investigate the potential role of eccentricity in the present findings.
